# Expression of UPR effector proteins ATF6 and XBP1 reduce colorectal cancer cell proliferation and stemness by activating PERK signaling

**DOI:** 10.1038/s41419-019-1729-4

**Published:** 2019-06-21

**Authors:** Claudia N. Spaan, Wouter L. Smit, Jooske F. van Lidth de Jeude, Bartolomeus J. Meijer, Vanesa Muncan, Gijs R. van den Brink, Jarom Heijmans

**Affiliations:** 1 Amsterdam UMC, University of Amsterdam, Department of Gastroenterology and Hepatology, Tytgat Institute for Liver and Intestinal Research, Meibergdreef 71, Amsterdam, The Netherlands; 2 Amsterdam UMC, University of Amsterdam, Department of Internal Medicine and Hematology, Meibergdreef 9, Amsterdam, The Netherlands; 3 Present Address: Roche Innovation Center Basel, F. Hoffmann-La Roche AG, Basel, Switzerland

**Keywords:** Colon cancer, Endoplasmic reticulum

## Abstract

The unfolded protein response (UPR) acts through its downstream branches, PERK-eIF2α signaling, IRE1α-XBP1 signaling and ATF6 signaling. In the intestine, activation of the UPR through the kinase PERK results in differentiation of intestinal epithelial stem cells and colon cancer stem cells, whereas deletion of XBP1 results in increased stemness and adenomagenesis. How downstream activation of XBP1 and ATF6 influences intestinal stemness and proliferation remains largely unknown. We generated colorectal cancer cells (LS174T) that harbor doxycycline inducible expression of the active forms of either XBP1(s) or ATF6^1-373^. Activation of either XBP1 or ATF6 resulted in reduced cellular proliferation and reduced expression of markers of intestinal epithelial stemness. Moreover, XBP1 and ATF6 activation reduced global protein synthesis and lowered the threshold for UPR activation. XBP1-mediated loss of stemness and proliferation resulted from crossactivation of PERK-eIF2α signaling and could be rescued by constitutive expression of eIF2α phosphatase GADD34. We thus find that enforced activation of XBP1 and ATF6 results in reduction of stemness and proliferation. We expose a novel interaction between XBP1 and PERK-eIF2α signaling.

## Introduction

The intestinal epithelium is a rapidly renewing tissue that is fueled by intestinal stem cells located at the bottom of the crypt. Intestinal stem cells are defined by the capability to self-renew, proliferate, and differentiate and are therefore regarded as critical drivers that renew the intestinal epithelial layer every 3–5 days^1^. Intestinal stem cells are not only important during homeostasis or after injury, but they are thought to be the cells of origin in the majority of colon cancers^2^. After acquiring consecutive mutations, mostly initiated by an inactivating mutation in the adenomatous polyposis coli (*APC*) gene, colon cancer stem cells arise from intestinal stem cells^3,4^. In contrast to differentiated tumor cells, these cancer stem cells are resistant to classical chemotherapy and novel strategies are emerging that aim at differentiating these cells in order to increase sensitivity to anticancer therapies^5,6^.

Previously, we found that intestinal stem cells differentiate upon activation of the unfolded protein response (UPR)^7^. The UPR is an evolutionarily conserved signaling pathway activated upon endoplasmic reticulum (ER) stress, which occurs when unfolded and misfolded proteins accumulate in the lumen of the ER^8,9^. Activation of the UPR in the intestinal epithelium results in decreased stemness. In addition, stem cells that are found in hyperproliferative crypts of mice that carry homozygous epithelial *Apc* mutations were equally differentiated by UPR activation^10^. Moreover, ER stress decreases tumor burden in mice carrying the heterozygous APC mutation^11^.

The UPR consists of three effector proteins: PKR like ER kinase (PERK), Inositol-requiring enzyme 1 (IRE1), and Activating Transcription Factor 6 (ATF6), which aim to resolve ER stress by transiently inhibiting global translation, expanding the ER capacity and upregulating important ER-resident chaperones, such as Grp78 and Grp94^12^.

PERK is activated upon ER stress and is one of the four kinases that phosphorylates the alpha unit of eukaryotic translation initiation factor 2(eIF2α)^13,14^. The strong binding of phosphorylated eIF2α to its GEF reduces levels of eIF2-GTP available for initiation of translation, resulting in reduced translation. Translation inhibition leads, among other things, to loss of cyclin D1 from cells, causing a G1 arrest^15^. Temporary inhibition of translation leads to a loss of short-lived proteins such as MYC^16,17^. Despite global inhibition of translation, a subset of *mRNAs* is selectively synthethized. These proteins include Activating transcription factor 4 (ATF4), C/EBP-homologous protein (CHOP) and Growth arrest and DNA damage-inducible 34 (GADD34), an eIF2α phosphatase^18^. These *mRNAs* usually contain an upstream open reading frame.

IRE1 is a kinase and endoribonuclease that senses unfolded proteins in the lumen of the ER via its N-terminal domain, which leads to activation by auto-phosphorylation. The endoribonuclease domain excises a 26-base-pair intron from the X-box-protein1 (*XBP1*) *mRNA*, which results in the *XBP1(s) mRNA*, encoding an active transcription factor^19^. XBP1(s) activity leads to expansion of the ER and is crucial for cellular differentiation, especially in different secretory cell types^20–24^. Previously, it was shown that mice that lack epithelial Xbp1 have increased stem cell numbers and adenomagenesis. In this study, the oncogenic effects of loss of Xbp1 were attributed to Ire1α and Stat3 signaling^25^.

ATF6 translocates to the Golgi apparatus upon ER stress. Site-1 protease and Site-2 protease cleave ATF6, whereupon the cytosolic transcription-factor is released^26,27^. Expression of the active ATF6, ATF6^1–373^, upregulates ER chaperones Grp78, and Grp94 and leads to expansion of the ER^26,28^.

We have previously shown that PERK-eIF2α signaling plays an important role in the decrease of intestinal stemness after ER stress. Moreover, XBP1 has been linked to a decrease in intestinal stemness^25^. It is not known, however, to what extent ATF6 and XBP1(s) affect intestinal stemness^29^. Because XBP1 and ATF6 have extensively overlapping sets of target genes, we set out to investigate both XBP1 and ATF6 signaling in colon cancer cells, and investigate the crosstalk of these branches with PERK- eIF2α^20,29^.

## Materials and methods

### DNA constructs and cloning of plasmids

*XBP1(s) mRNA* was amplified from a human cDNA pool using primers gatgcccagagaaccgtgaaagtg and cctcactttgtaatacactttcc. The truncated *ATF6*^1–373^ encoding the transcriptionally active part of ATF6 was a kind gift from Mori and coworkers^26^. *XBP1(s)* and *ATF6*^1–373^ were cloned into the pRetroTight vector (Clontech). The constitutively active hamster Gadd34 (also termed A1) was a kind gift from Ron and coworkers^30^. This coding sequence was subcloned into the pCDNA 3.1 (+) vector (Invitrogen). Lentiviral shRNA constructs for *PERK* were obtained from the Mission shRNA library (Sigma).

### Cell culture and generation of stable cell lines

LS174T colorectal cancer cells (ATCC CL188) and SW480 colorectal cancer cells (ATCC CCL228) were grown in Dulbecco’s modified Eagle’s medium with 10% fetal calf serum (FCS) and 1% penicillin/streptomycin under standard culture conditions. DLD-1 colorectal cancer cells (ATC CCL-221) were grown in RPMI with 10% FCS and 1% penicillin/streptomycin under standard culture conditions.

To generate cells that could inducibly express ATF6^1–373^ and XBP1(s), we used the pRetro-tight system (Clontech). We transduced cells with viral particles containing pLenti-CMV-rtTA3 (Addgene plasmid # 26429) and cultured cells in medium containing 5 μg/L blasticidin. Subsequently cells were transduced retrovirally with XBP1(s) or ATF6^1–373^ constructs and cultured in medium containing 10 μg/ml puromycin. Stable transduction of cells containing the lentiviral pLKO_shPERK was achieved by transducing lentiviral particles into cells according to standard protocols. To generate cells that harbored stable expression of the constitutively active hamster Gadd34 (^*CA*^*Gadd34*), the pCDNA3.1-^CA^Gadd34 construct was transfected in indicated cells using polyethyleneimine. Approximately 10E4 cells were seeded in 145 cm^2^ culture dishes and cultured with growth medium supplemented with 600 μg/ml G418 (Gibco™ LS10131027). After ~2 weeks, colonies were picked and clonally expanded for further experiments. All experiments were performed after 18 h of induction with doxycycline (1 μg/ml) unless specifically otherwise stated.

### Crystal violet

For crystal violet analyses, cells were fixed with 4% paraformaldehyde in PBS for 15 min and subsequently immersed in 5 mg/ml crystal violet (Sigma C3886) in 2% ethanol in H_2_O.

### EdU incorporation

For measurement of EdU incorporation in cells, we used Click-iT® EdU Alexa Fluor® 647 (Thermofisher C10634) according to the manufacturer’s protocol. The experiments were performed on the LSR Fortessa flow cytometer. Results were analyzed with FlowJo V10 software.

### ER tracker

For analyses of the ER size, cells were plated on round 18 mm coverslips in a 12-well plate and stimulated with doxycycline 1 μg/ml after adhering. Cells were stained with ER-Tracker™ Red (BODIPY™ TR Glibenclamide, Thermofisher #E34250) and briefly fixated with 4% paraformaldehyde in PBS. Cells were imaged using confocal microscopy and ER surface was quantified using ImageJ software.

### Immunoblot

For western blot analysis, cells were lysed in lysisbuffer (Cell Signaling) containing Protease Inhibitor Cocktail (Roche #13538100), and sonicated. Samples were run on sodium dodecyl sulfate polyacrylamide gel electrophoresis gels under reducing conditions and transferred to a PVDF membrane. Specific detection was done by incubating the blot overnight in TBS with 0.1% Tween 20 with 1% BSA. Antibodies used for detection were anti-PERK (Cell Signaling #3192, 1:1000), anti-BiP (Cell Signaling #3183, 1:1000), anti-phospho-eIF2α (Cell Signaling #3398, 1:1000), anti-CHOP (Cell Signaling #2895, 1:1000), anti-XBP1 (Santa Cruz 7160, 1:500), Anti-eIF2α (Cell Signaling #2103, 1:1000), anti-c-Myc (Santa Cruz #764, 1:1000), anti-ATF6 (Bioadacemia 73–500, 1:1000), anti-IRE1α (Cell Signaling #3294, 1:1000), and anti-beta actin (Sigma, A1978, 1:100,000). Secondary antibody detection with HRP labeled polyclonal antibodies was performed (Dako, Goat Anti-Rabbit #P0448, Goat Anti-Mouse #P0447, 1:2000), and antibody visualization was with Lumilight Plus (Roche,12015196001).

### 35S-methionine incorporation assay

To measure global protein synthesis rates, we quantified the incorporation of ^35^S-labeled methionine and cysteine into newly translated proteins. Cells were exposed to 15 min methionine starvation followed by a 45 min methionine incubation to label newly synthesized proteins, using 1 µl (1.25 µCi/ml) of EasyTag™ L-[35 S]-Methionine (PerkerElmer®) per well at 37 C, 95% humidity and 5% CO_2_ conditions. After labeling, cells were washed twice in cold PBS, harvested and centrifuged in cold PBS to remove supernatant. Next, cell pellets were lysed in cell lysis buffer (Cell Signaling). Lysate was blotted on labeled 24 mm glass microfiber filters (GF/C Whatman®) that were presoaked in 20% TCA. Filters were placed in a vacuum manifold and incubated in 10% ice-cold TCA for 15 min, followed by 10% TCA at 90–95 °C for 10 min. Filters were washed twice with cold 2% TCA and then twice with 95% ethanol to remove TCA. Next, filters were air dried for 1 h at room temperature and placed in liquid scintillation cocktail (Ultima Gold, PerkerElmer®). Radioactivity was quantified using a scintillation counter (Tri-Carb 2900TR).

### RNA extraction and quantitative RT-PCR

For RNA purification, cells were lysed after 18 h of treatment with doxycycline (1 μg/ml) in 1 ml TRI Reagent® (Sigma T9424). Further RNA purification was according to manufacturers’ instructions. For cDNA synthesis, 1 μg of RNA was transcribed using RevertAid reverse transcriptase (Thermo Scientific) using DNA hexamers of randomized sequence (Promega). Quantitative RT-PCR was performed using SensiFAST™ polymerase mix (Bioline 98020) according to manufacturers’ protocol on a BioRad CFX96 Touch™ Real-Time PCR detection system using specific primers for the *mRNA* of interest. RT-PCR primers (all human) were *GAPDH* forward 5′-AAGGTGAAGGTCGGAGTCAA-3′ reverse 5′-AATGAAGGGGTCATTGATGG-3′, *XBP1(s)* forward 5′- CCGCAGCAGGTGCAGG-3′ reverse 5′-GAGTCAATACCGCCAGAATCCA-3′, *ATF6*^*1–373*^ forward 5′- GCCTTTATTGCTTCCAGCAG-3′ reverse 5′-TGAGACAGCAAAACCGTCTG-3′, *LUCIFERASE* (firefly) forward 5′-TTACACCCGAGGGGGATGAT-3′ reverse 5′-CCAGATCCACAACCTTCGCT-3′, *GRP78* forward 5′-CATCACGCCGTCCTATGTCG-3′ reverse 5′-CGTCAAAGACCGTGTTCTCG-3′, *HSP90B1* (*GRP94)* forward 5′-TGTAATTGCTGACCCAAGAGG-3′ reverse 5′-TCCAATTCAAGGTAATCAGATGC-3′, *DDIT3(CHOP)* forward 5′-AGCCAAAATCAGAGCTGGAA-3′ reverse 5′-TGGATCAGTCTGGAAAAGCA-3′, *PERK* forward 5′-TCATCCAGCCTTAGCAAACC-3′ reverse 5′-ATGCTTTCACGGTCTTGGTC-3′, *DNAJB9 (ERDJ4)* forward 5′- CAGCTCTTGTGGAGGAGCAG-3′, *ATF4* forward 5′-CAGCAGCACCAGGCTCT-3′ reverse 5′-TCGAAGGTGTCTTTGTCGGT-3′, *EDEM1* forward 5′- GCTCAACCCCATCCACTG

-3′ reverse 5′-CCAATGCATCAACAAGAGTCA-3′, *DNAJC3 (P58IPK)* forward 5′- ACAAGGAAAACTTGATGAAGCAG-3′ reverse 5′-TGAGACTGTGCTTCCTTTTCTTC-3′, *DNAJB11 (HEDJ)* forward 5′-GGATCTGGGTGCTGCTTATG-3′ reverse 5′-TGTCTCCATGGGAGCTCTG-3′, *ASCL2* forward 5′-GGCACCAACACTTGGAGATT-3′ reverse 5′-CCCTCCAGCAGCTCAAGTTA-3′, *AXIN2* forward 5′-TCTGGTGCAAAGACATAGCCA-3′ reverse 5′-AGTGTGAGGTCCACGAAAC-3′, reverse 5′- AATGCAGATTGCAAAGATGAAA-3′, *LGR5* forward 5′-GTTTCCCGCAAGACGTAACT-3′ reverse 5′- CAGCGTCTTCACCTCCTACC-3′, *OLFM4* forward 5′-GTGGACAGAGTGGAACGCTT-3′ reverse 5′- CACACTAATTAATTGGACATATTCCCT-3′, *ha_GADD34* forward 5′-CCACCTGGAAGAGAGAGTGC-3′ reverse 5′-GGGATCAGCTGAGAAAGACG-3′, *BMI1* forward 5′-CGTGTATTGTTCGTTACCTGGA-3′ reverse 5′-TTCAGTAGTGGTCTGGTCTTGT-3′, *LRIG* forward 5′-CTGGACGCGGAGCCTAAAC-3′ reverse 5′-TGTAGGTTCGGCAAGTCCTCA-3′, *HOPX* 5′-GACAAGCACCCGGATTCCA-3′ reverse 5′-GTCTGTGACGGATCTGCACTC-3′, *TERT* forward 5′-TCACGGAGACCACGTTTCAAA-3′ reverse 5′-TTCAAGTGCTGTCTGATTCCAAT-3′.

### Statistics

Statistical analyses were performed using GraphPad Prism software (Graphpad, La Jolla, CA). All data are presented as mean ± SEM of three independent experiments of technical triplicates. For comparison of two groups, Student’s *t*-test was used, for grouped analyses, two-way ANOVA was used followed by Bonferroni’s post-hoc test for multiple comparisons. *P* value of < 0.05 was deemed significant.

## Results

### Activation of XBP1(s) and ATF6 results in upregulation of UPR target genes

We set out to examine effects of UPR transcription factors XBP1 and ATF6 on intestinal epithelium. To this end, we utilized LS174T colorectal cancer cells that harbor mutations in the WNT signaling pathway, causing them to resemble cells of the intestinal crypt. These cells have the possibility to differentiate and express markers of terminally differentiated cells of the intestine, such as mucins and cyclin dependent kinase inhibitor P21^31,32^. We transduced LS174T cells with constructs enabling doxycycline inducible expression of transcription factors XBP1 and ATF6. Under homeostatic conditions, levels of ER stress are low and transcripts of XBP1 and ATF6 encode for proteins that are not transcriptionally active. We therefore generated cells that carried tetracycline inducible transcripts of active XBP1 (XBP1(s)) and of the transcriptionally active cytosolic domain (amino acids 1 to 373) of ATF6 (further referred to as ATF6^1–373^)^26^.

**Fig. 1:**
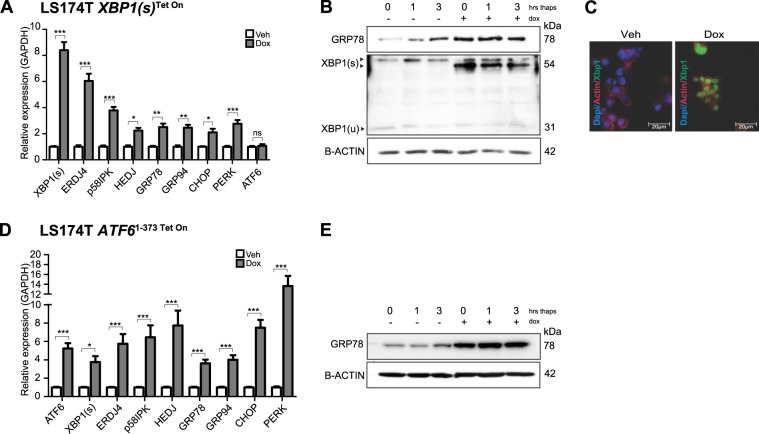
LS174T cells expressing XBP1(s) or ATF6^1–373^ upregulate general UPR target genes. **a** Quantitative RT-PCR analysis for *XBP1(s)* and downstream target genes upon induction of XBP1(s) expression. **b** Protein level of XBP1(s) and GRP78 in LS174T *XBP1(s)*^Tet On^ cells. Cells were incubated for 0, 1 or 3 h with thapsigargin 200 nM. Note that transgenic XBP1(s) had a slightly lower molecular weight than endogenous XBP1(s). **c** Immunostaining of XBP1 in LS174T *XBP1(s)*^Tet On^ cells. **d** Quantitative RT-PCR analysis for *ATF6*^*1–373*^ and downstream target genes upon induction of ATF6^1–373^ expression. **e** Protein level of GRP78 in LS174T *ATF6*^1–373 Tet On^ cells. Cells were treated with vehicle or doxycycline 1 μg/mL for 18 h. All data are shown as means ± SEM. *P < 0.05, **P < 0.01, ***P < 0.001

Using quantitative PCR, we confirmed expression of XBP1(s) and found it harbored transcriptional activity, as judged by increased expression of direct target genes *P58*^*IPK*^, *ERDJ4*, ER chaperones *GRP78*, *GRP94* and UPR transcription factor *CHOP* (Fig. 1a)^29^. Among upregulated genes, we found that UPR effector *PERK* was upregulated 2.8-fold, confirming previous reports that identify *PERK* as a direct transcriptional target of XBP1(s)^23^.

Immunoblots of XBP1(s) cells confirmed high expression of XBP1(s), which reproducibly had a slightly lower molecular weight than endogenous XBP1(s). XBP1(s)-expressing cells exhibited upregulation of target chaperone GRP78. Expression of XBP1(s) and GRP78 were upregulated to a similar extent as we observed in control cells after treatment with thapsigargin, a chemical inducer of ER-stress (Fig. 1b and Supp. Fig. 1A). It has been reported that IRE1α protein levels increase upon deletion of XBP1^25^. We found no altered expression in IRE1α protein level upon enforced expression of XBP1(s), while thapsigargin treatment increased the protein levels as expected. We additionally confirmed that expression of transcription factor XBP1(s) was indeed localized in the nucleus (Fig. 1c). It has been established that XBP1(s) signaling increases the capacity of the ER by inducing ER-biogenesis^20^. We therefore next analyzed the volume of the ER upon induction of XBP1(s). Indeed, we found that enforced expression of XBP1(s) resulted in increased size of the ER to a similar extent as we observed after treatment with thapsigargin (Supp Fig. 1B).

Parallel to XBP1(s) expression, ATF6 expression is linked to ER expansion and upregulation of ER chaperones^26,28^. Similar to our observations in XBP1(s)-expressing cells, we confirmed upregulation of ATF6^1–373^ protein upon induction with doxycycline in ATF6^1–373^ cells. Upregulation of ATF6^1–373^ and GRP78 was to a similar extent as in control cells treated with thapsigargin (Supp. Fig. 1C, D, E). Furthermore, IRE1α levels were not grossly altered upon ATF6^1–373^ expression. In addition, we confirmed transcriptional activity of ATF6^1–373^ in stably transfected LS174T cells. Moreover, we observed expected upregulation of UPR target genes on *mRNA* and protein level upon ATF6^1–373^ overexpression (Fig. 1d, e).

ATF6^1–373^-expressing cells had a similar increase in ER size (Supp. Fig. 1F). Although *PERK mRNA* is increased by direct binding of XBP1 to the PERK promoter, this has not been demonstrated for ATF6. We did, however, observe that expression of ATF6^1–373^ resulted in 13-fold upregulation of *PERK mRNA*.

These results verify that overexpression of XBP1(s) or ATF6^1–373^ results in upregulation of UPR target genes and increased size of the ER.

### Expression of XBP1(s) and ATF6^1–373^ results in reduced stemness and proliferation

**Fig. 2:**
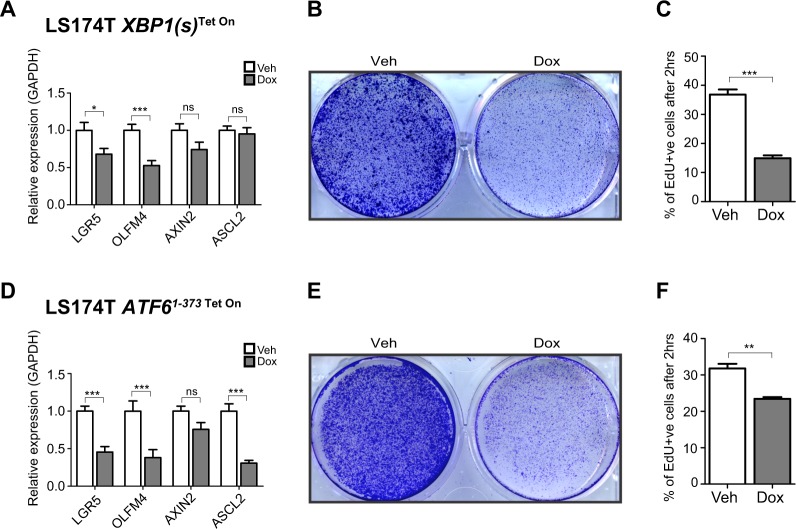
XBP1(s) or ATF6^1–373^ expression results in reduced stemness and cell proliferation in LS174T colorectal cancer cells. **a** Quantitative RT-PCR analysis for intestinal stem cell markers *LGR5, OLFM4* and *ASCL2* or Wnt target genes *LGR5* and *AXIN2* in LS174T *XBP1(s)*^Tet On^ cells. **b** Crystal violet cell viability assay in LS174T *XBP1(s)*^Tet On^ cells. **c** FACS-based EdU incorporation assay in LS174T *XBP1(s)*^Tet On^ cells; the assay was performed after 2 h of EdU incorporation. **d** Quantitative RT-PCR analysis for intestinal stem cell markers *LGR5, OLFM4* and *ASCL2* or Wnt target genes *LGR5* and *AXIN2* in LS174T *ATF6*^*1–373* Tet On^ cells. **e** Crystal violet cell viability assay in LS174T *ATF6*^*1–373* Tet On^ cells. **f** FACS-based EdU incorporation assay in LS174T *ATF6*^*1–373* Tet On^ cells; the assay was performed after 2 h of EdU incorporation. All data are shown as means ± SEM. *P < 0.05, **P < 0.01, ***P < 0.001

We have previously shown that general UPR activation results in PERK-dependent reduction of intestinal epithelial stemness^7^. Moreover, mice that lack UPR transcription factor *Xbp1* in the intestinal epithelium display increased intestinal stem cell numbers^25^. We next examined stemness in cells that had enforced expression of XBP1(s). To this end we performed quantitative RT-PCR for intestinal stem cell marker genes *LGR5* and *OLFM4* and found that these were significantly decreased upon induction of XBP1(s) (Fig. 2a). Stem cell genes LGR5 and OLFM4 mark crypt base columnar stem cells that harbor high WNT signaling. In past years, a number of stem cell markers have been described that mark alternatively localized stem cells, which are not driven by WNT signaling, including BMI1, LRIG, HOPX and TERT^33–36^. We examined *mRNA* expression of these stem cell markers, and found only TERT to be reduced upon induction of both XBP1(s) and ATF6^1–373^. Other markers were unaltered of even slightly elevated (Supp. Fig. 2A and B). These results show that in LS174T cells, which are cells with high WNT signaling, stem cell markers that are driven by WNT signaling are reduced upon expression of XBP1(s) and ATF6^1–373^, whereas WNT independent alternative stem cell markers are largely unaffected.

Additionally, XBP1(s) expression resulted in significant decrease in cell numbers (Fig. 2b). This was, at least in part, accounted for by a decrease in cellular proliferation of 60% as measured by EdU incorporation (Fig. 2c). Similar to XBP1(s)-expressing cells, ATF6^1–373^ expression resulted in decreased cellular proliferation and decreased expression of stem cell markers (Fig. 2d, e). Moreover upon ATF6^1–373^ expression, EdU incorporation was decreased (Fig. 2f).

We have previously shown that induction of UPR activity may result in loss of stemness through rapid loss of short lived transcription factors that are critical for the stem cell phenotype (such as C-MYC) we tested whether expression of C-MYC was altered upon XBP1(s) induction^7,37^. Indeed, we found markedly reduced expression of C-MYC protein after induction of XBP1(s) (Supp. Fig. 2C).

To exclude a cell line specific effect of the observed growth disadvantage upon expression of XBP1(s) or ATF6^1–373^, we generated two alternative colorectal cancer cell lines harboring inducible expression of XBP1(s) and ATF6^1–373^ (SW480 and DLD-1, Supp. Fig. 3A). Similar to what we observed in LS174T cells, Expression of either XBP1(s) or ATF6^1–373^ resulted in reduced cell viability (Supp. Fig. 3B). We could however not detect any *mRNA* of *OLFM4* in both these cell lines, and SW480 cells did not express *mRNA* of *LGR5* and therefore continued experiments with LS174T cells solely.

To rule out ER stress and subsequent UPR activation through high upregulation of transgenic proteins or through heterotopic actions of doxycycline, we generated control cells expressing high levels of firefly luciferase in a doxycycline responsive manner (LS174T Luciferase^TetOn^). In these cells we observed no effects on expression of UPR target genes or stem cell markers as well as unaltered growth (Supp. Fig. 3C–H).

Thus, enforced expression of XBP1(s) and ATF6^1–373^ in colon cancer cells results in decrease in stemness and proliferation, not attributable to doxycycline.

### Activation of XBP1(s) and ATF6^1–373^ enhances sensitivity to UPR activation

Enforced expression of XBP1(s) or ATF6^1–373^, even in the absence of ER stress, increased the size of the ER and the expression of ER chaperones. ER chaperones such as GRP78 are needed for proper folding of unfolded proteins to restore ER homeostasis. We therefore hypothesized that a higher amount of chaperones and an increased ER size, resulting from transgenic expression of XBP1(s) or ATF6^1–373^, could result in increased resistance to ER stress. We treated cells with a very low level of thapsigargin intended to yield no growth impairment in control cells. To our surprise, cells with enforced expression of XBP1(s) or ATF6^1–373^ had reduced cell viability after a short pulse exposure to these low levels of thapsigargin (Supp. Fig. 4A, B). These results indicate that enforced expression of XBP1(s) or ATF6^1–373^ reduces the threshold for activation of the UPR and enhances sensitivity to ER stress.

### Expression of XBP1(s) and ATF6^1–373^ causes activation of PERK and inhibition of global protein synthesis

**Fig. 3:**
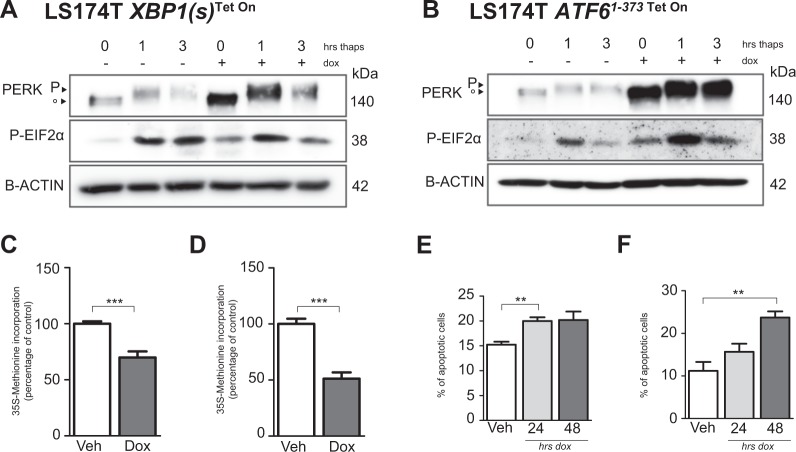
XBP1(s) or ATF6^1–373^ expression upregulates PERK-eIF2α and inhibits global translation. **a**, **c**, **e** LS174T *XBP1(s)*^Tet On^ cells. **b**, **d**, **f** LS174T *ATF6*^*1–373* Tet On^ cells. **a**, **b** Protein levels of PERK and phosphorylated EIF2α. Cells were incubated for 0, 1 or 3 h with thapsigargin 200 nM. **c**, **d** Global protein translation rate, measured by ^35S^Methionine incorporation assay. **e**, **f** Percentage of apoptotic cells, measured with propidium iodide and Annexin V staining on Flow Cytometry. All data are shown as means ± SEM. *P < 0.05, **P < 0.01, ***P < 0.001

Our observations of growth reduction and increased expression of *PERK mRNA* in XBP1(s) and ATF6^1–373^-expressing cell lines may result from downstream activation of PERK. We therefore analyzed activity of PERK upon induction of XBP1(s) or ATF6^1–373^. Indeed, PERK protein was upregulated and downstream phosphorylation of eIF2α was observed (Fig. 3a and B). Phosphorylation of eIF2α results in a transient inhibition of global protein translation^13^. We therefore analyzed cellular global protein synthesis using radioactively labeled 35S-methionine. Expression of XBP1(s) and ATF6^1–373^ resulted in a significant decrease in protein synthesis (Fig. 3c, d)^38^. To study the kinetics by which induction of XBP1(s) and ATF6 resulted in eIF2α phosphorylation and loss of markers of stemness, we treated cells for different durations with doxycycline. Interestingly, we found that phosphorylation of eIF2α occurred slightly earlier upon ATF6 induction than upon XBP1(s) induction (Supp. Fig. 5A, B). In addition, decrease of LGR5 *mRNA* was incremental over time in both cell lines (Supp. Fig. 5C, D).

Previous reports have shown that high and prolonged CHOP signaling, downstream of phosphorylated eIF2α, leads to apoptosis^39–41^. We therefore determined whether prolonged XBP1(s) or ATF6^1–373^ signaling, with established activation of PERK- phospho-eIF2α, led to an increase in apoptotic cells. We measured both early and late apoptosis with Propidium Iodide (PI) and Annexin V staining on flow cytometry. The number of apoptotic cells was increased by 31% after induction of XBP1(s) (Fig. 3e). In the ATF6^1–373^-expressing cells, apoptosis was increased with 39% (Fig. 3f).

We conclude that activation of XBP1(s) and ATF6^1–373^ is capable of activating PERK-eIF2α signaling, which might account for reduced global protein synthesis rates and induced apoptosis in these cells.

### XBP1(s) and ATF6^1–373^ activation results in cell cycle arrest in distinct phases

It has been described that UPR activation through PERK signaling can result in cell cycle arrest in both G1 and G2 phases. G1 arrest is thought to be mediated by Cyclin D1 abrogation, whereas G2 arrest results from Chk1 effector kinase activation^15,42,43^. To further elucidate how XBP1(s) and ATF6^1–373^ expression resulted in growth arrest, we performed cell cycle analysis using Propidium Iodide. Similar to thapsigargin treatment, XBP1(s) expression arrested cells in the G1 phase (Supp. Fig. 6A)^15^. Interestingly, ATF6^1–373^-expressing cell lines upregulated PERK-eIF2α to the same extent, though ATF6^1–373^ expression arrested cells in the G2 phase (Supp Fig. 6B).

### XBP1(s)-induced growth arrest is mediated by PERK-eIF2α signaling

**Fig. 4:**
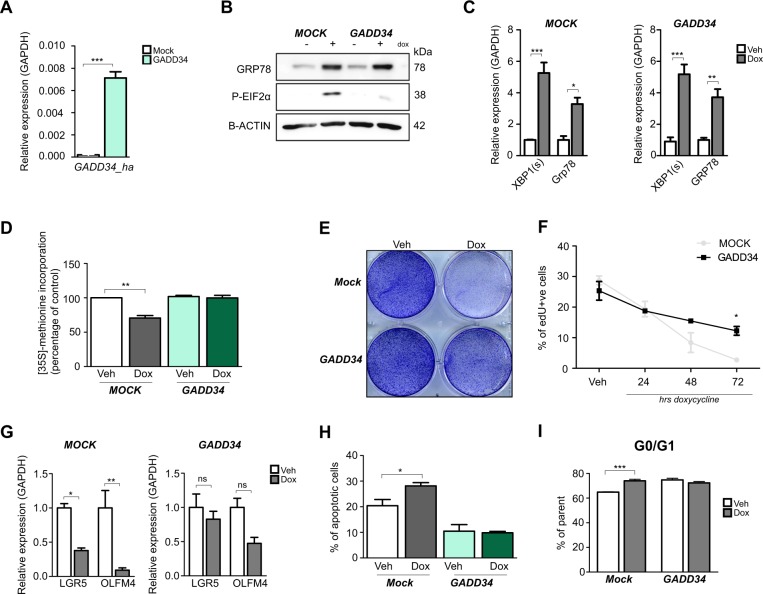
Inhibition of eIF2α phosphorylation rescues XBP1(s) induced growth arrest. **a** Quantitative RT-PCR analysis for hamster *GADD34* in cells expressing either a mock or a GADD34 vector. **b** Protein levels of GRP78 and phosphorylated eIF2α in LS174T *XBP1(s)*^Tet On^ cells expressing either a mock or a GADD34 vector. **c** Quantitative RT-PCR analysis for *XBP1(s)* and downstream target genes upon induction of XBP1(s) expression in the presence of either a mock or a GADD34 vector. **d** Global protein translation rate, measured by ^35S^Methionine incorporation assay. **e** Crystal violet cell viability assay in LS174T *XBP1(s)*^Tet On^ cells. **f** FACS-based EdU incorporation assay in LS174T *XBP1(s)*^Tet On^ cells; assay was performed after 2 h of EdU incorporation. **g** Quantitative RT-PCR analysis for intestinal stem cell markers *LGR5* and *OLFM4*. **h** Percentage of apoptotic cells, measured with propidium iodide and Annexin V staining on Flow Cytometry. (**i**) Quantification of cell cycle analysis performed on Flow Cytometry using propidium iodide. All data are shown as means ± SEM. *P < 0.05, **P < 0.01, ***P < 0.001

Previously, we have shown that PERK-eIF2α signaling is important for the decreased stemness that is observed in colorectal cancer cells after induction of ER stress. Moreover, reversing eIF2α phosphorylation by continuous dephosphorylation of eIF2α using GADD34 was capable of rescuing loss of markers of stemness in these cells^7,30^. To further implicate PERK-eIF2α signaling in XBP1(s) and ATF6^1–373^ induced growth retardation, we transduced cells with lentiviral particles containing short hairpin RNA fragments (*shRNA*) directed against *PERK*. In LS174T-XBP1(s)^Tet On^**-***shPERK* and LS174T-ATF6^1–373Tet On^**-***shPERK* cells, PERK expression was significantly reduced on both *mRNA* and protein levels (Supp Fig. 7A–D). Downstream phosphorylation of eIF2α was however only mildly reduced and downstream activation of eIF2α targets *ATF4* and *CHOP* were not inhibited. Reduced cell viability upon XBP1(s) or ATF6^1–373^ expression was unaltered in cells expressing *PERK shRNA* (Supp Fig. 7E, F). Lack of inhibition of PERK targets may be accounted for by activation of redundant eIF2α-kinase upon induction of ER-stress^38^. To overcome redundancy, we generated XBP1(s) and ATF6^1–373^-expressing cells that stably expressed high levels of a constitutively active form of GADD34 (Fig. 4a). Interestingly, in LS174T-ATF6^1–373^ cells, transgenic GADD34 expression abated over a short period of time in multiple distinct clonally grown cell lines and eIF2α phosphorylation was not satisfactorily inhibited. We could however generate LS174T-XBP1(s)^Tet On^**-**GADD34 cells in which only very modest levels of eIF2α phosphorylation could be observed upon induction of XBP1(s) (Fig. 4b), though increase in *XBP1(s) mRNA* and its downstream UPR target genes was similar to observed in the parental XBP1(s)-expressing cell line (Fig. 4c). In addition, GADD34 expression did not alter GRP78 protein levels. We found that in LS174T**-**XBP1(s)^Tet On^**-**GADD34 cells, reduced global protein synthesis, as observed in XBP1(s) cells was fully rescued (Fig. 4d). In addition, in LS174T**-**XBP1(s)^Tet On^**-**GADD34, reduced proliferation was largely rescued (Fig. 4e, f). Moreover, stem cell markers were largely normalized upon induction of XBP1(s) in these cells (Fig. 4g). Incomplete normalization of stem cell markers may have resulted from remaining modest levels of phosphorylated eIF2α. Potentially, rescue of XBP1(s) induced growth arrest in GADD34-expressing cells resulted from decreased apoptosis (Fig. 4h). Interestingly, cell cycle arrest in G0/1 upon doxycycline could be rescued in the GADD34-expressing cells, though already a larger percentage of unstimulated cells expressing GADD34, were in the G0/1 phase of the cell cycle (Fig. 4i). These results suggest that XBP1(s)-induced effects on cellular proliferation, translation and apoptosis are mediated by PERK-eIF2α signaling.

## Discussion

The UPR is a signaling pathway that controls cellular homeostasis at the level of protein quality. Accumulating evidence points towards a role of the UPR in cell fate decisions such as differentiation of intestinal epithelial stem cells and colorectal cancer cells. We investigated differential roles for distinct UPR components in these processes.

It has previously been shown that activation of PERK-eIF2α signaling results in reduced proliferation and increased apoptosis^44^. Moreover, we have previously implicated this pathway in loss of intestinal epithelial stemness and enforced differentiation^7^. Our current experiments show that activation of the two alternative branches of the UPR, XBP1 and ATF6, also results in reduced proliferation and diminished stemness. We have corroborated reduced cell viability experiments in three cell lines, leading us to think that this phenomenon is not cell-line specific. These results are in line with studies showing that epithelial loss of Xbp1 in mice resulted in increased stem cell numbers and increased tumorigenesis^25^. Moreover, increased apoptosis upon ATF6 expression in chondrocytes and myoblasts corroborates these findings^45,46^. In contrast, two studies report pro-tumorigenic properties of cells upon overexpression of XBP1 or ATF6^47,48^. These effects of XBP1 and ATF6 may result from expression in alternative cell types or stable expression of XBP1 and ATF6 instead of inducible expression, which may have resulted in selection of cells.

Our findings that XBP1 and ATF6 expression result in reduced proliferation and stemness are further strengthened by the fact that these cells exhibit reduced global protein synthesis. In a previous report, it was shown that XBP1(s) and ATF6^1–373^ expression in HEK293T cells did not result in reduced protein production capacity. In these reports, a small but non-significant reduction in 35S incorporation was noted in the XBP1(s) expressing HEK293T cells after only a short induction period of 12 h. Discrepancy with significant reduction in 35S incorporation in our experiments may depend on this short induction period, potentially preceding significant reduction of translation^49^.

LS174T cells that express XBP1 or ATF6 exhibit increased sensitivity to thapsigargin. Interestingly, in most models examining either gain or lack of activity of the principal UPR components, sensitivity to ER stress increased. This was shown for PERK^−/−^ mouse embryonic stem cells, homozygous or heterozygous knockout of Xbp1 and loss of Atf6^50–52^. ATF6^−/−^ and IRE1α^−/−^ melanoma cells showed increased sensitivity to ER stress in the presence of adequate PERK signaling. Likely, balance in the UPR is regulated to such an extent that perturbation of any UPR component may result in increased sensitivity to ER stress, resulting in reduced viability.

Colorectal cancer cells that harbor XBP1 or ATF6 activation have a highly similar phenotype, potentially resulting from the combined transcriptional activity of these factors as heterodimers^53^. We find remarkable differences, however, in alteration of the cell cycle phase upon XBP1 or ATF6. Although it has been advocated that PERK activation can both lead to G1 and G2 arrest via different mechanisms, the differences between XBP1 induced G1 arrest and ATF6 induced G2 arrest remain to be elucidated^15,42,43^.

XBP1 and ATF6 both activate PERK-eIF2α signaling. Interestingly, phenotypical changes upon XBP1 induction, such as reduced growth, cell cycle arrest, and reduced global protein synthesis, result from reduced eIF2α signaling, since a rescue of this phenotype resulted from dephosphorylation of eIF2α.

Our experiments uncover a novel cross-interaction between activated XBP1 and ATF6 and PERK-eIF2α. We uncover that the interaction between XBP1 and PERK-eIF2α is mechanistically responsible for the antiproliferative phenotype of XBP1, which can be rescued. Our data show again the importance of PERK-eIF2α in cellular viability and stemness. These results may be utilized to target PERK-eIF2α in treatment or prevention of intestinal malignancies.

## Supplementary information

Supplemental figures legends

Supp. Fig. 3

Supp. Fig. 1

Supp. Fig. 2

Supp. Fig. 4

Supp. Fig. 5

Supp. Fig. 6

Supp. Fig. 7
