# Supplementary material for: Expression of UPR effector proteins ATF6 and XBP1 reduce colorectal cancer cell proliferation and stemness by activating PERK signaling
Source: Cell Death Dis. 2019 Jun 21;10(7):490. doi: 10.1038/s41419-019-1729-4 (PMC6588629; doi:10.1038/s41419-019-1729-4)
Supplement: Supplementary file 1 — Supplemental figures legends [file 41419_2019_1729_MOESM1_ESM.docx]

**SUPP FIG 1** Effect of XBP1(s) and ATF6^1-373^ expression on endoplasmic reticulum size. (A) Protein level of GRP78 and IRE1α in LS174T *XBP1(s)*^Tet On^ cells. Cells were incubated for 0, 3, 8 or 16 hours with thapsigargin 200nM in the presence of vehicle or doxycycline 1ug/mL. (B) ER tracker staining on LS174T *XBP1(s)*^Tet On^ cells after treatment with vehicle (veh), doxycycline 1ug/mL for 18 hours(dox) or thapsigargin 200nM (thaps). (C) Detection of pATF6 precursor (P) and its cleaved product (N) in LS174T *ATF6^1-373^* ^Tet On^ cells stimulated with doxycycline 1ug/mL for 2, 4 or 8 hours. (D) Detection of pATF6 precursor (P) and its cleaved product (N) in LS174T cells treated with thapsigargin 200nM for 3 or 8 hours. (E) Protein level of GRP78 and IRE1α in LS174T *ATF6^1-373^* ^Tet On^ cells. Cells were incubated for 0, 3, 8 or 16 hours with thapsigargin 200nM in the presence of vehicle or doxycycline 1ug/mL. (F) LS174T ATF61-373Tet On cells after treatment with vehicle, doxycycline or thapsigargin. All data are shown as means ± SEM. *, P < 0.05 (by Student’s t test).

**SUPP FIG 2** Expression of non-WNT driven alternative stem cell markers and C-MYC protein upon XBP1(s) and ATF6^1-373^ expression. (A,B) Quantitative RT-PCR analysis for alternative stem cell markers *BMI1, LRIG, HOPX* and *TERT* after 18 hours of doxycycline or vehicle. All data are shown as means ± SEM. *, P < 0.05. stimulation. (C) Protein level of

C-MYC and XBP1(s). Note that transgenic XBP1(s) had a slightly lower molecular weight than endogenous XBP1(s).

**SUPP FIG 3** Induction of UPR target genes and reduction of cell viability results from enforced expression of *XBP1(s)* or *ATF6^1-373^*. (A) Quantitative RT-PCR analysis for target genes in DLD-1 and SW480 cells expressing *XBP1(s)* or *ATF6^1-373^*. (B) Crystal violet cell viability assay in DLD-1 or SW480 cells expressing either doxycycline inducible *XBP1(s)* or *ATF6^1-373^*. Control LS174T cells with inducible Luciferase show no alterations in UPR target gene expression or cell viability. (C) Quantitative RT-PCR analysis for *Luciferase* and UPR target genes upon induction of Luciferase expression. (D) Protein level of GRP78 and phosphorylated EIF2α in LS174T *Luciferase*^Tet On^ cells. Cells were incubated for 0, 1 or 3 hours with thapsigargin 200nM. (E) Quantitative RT-PCR analysis for intestinal stem cell markers *LGR5, OLFM4* and *ASCL2* or Wnt target genes *LGR5* and *AXIN2* in LS174T *Luciferase*^Tet On^ cells. (F) Crystal violet cell viability assay in LS174T *Luciferase*^Tet On^ cells. (G) FACS based EdU incorporation assay in LS174T *Luciferase*^Tet On^ cells, assay was performed after 2hrs of EdU incorporation. (H) Quantification of cell cycle analysis using propidium iodide. All data are shown as means ± SEM. *, P < 0.05, **, P < 0.01, ***, P < 0.001.

**SUPP FIG 4** LS174T cells expressing XBP1(s) or ATF6^1-373^ exhibit increased sensitivity to extra ER stress. (A) LS174T *XBP1(s)*^Tet On^ cells were treated with a short pulse (0,5hr 50nM) of thapsigargin in the presence or without doxycycline 1ug/mL and a Crystal Violet cell viability assay was performed after 6 days. (B) LS174T *ATF6^1-373^* ^Tet On^ cells were treated with a short pulse (0,5hr 50nM) of thapsigargin in the presence or without doxycycline 1ug/mL and a Crystal Violet cell viability assay was performed after 6 days.

**SUPP FIG 5** Temporal kinetics of PERK-eIF2α signaling after enforced expression of XBP1(s) or ATF6^1-373^. (A,C) LS174T *XBP1(s)*^Tet On^ cells. (B,D) LS174T *ATF6^1-373^* ^Tet On^ cells. (A,B) Protein level of PERK, GRP78 and phosphorylated eIF2α in cells treated with different durations of doxycycline 1ug/mL. (C,D) Quantitative RT-PCR analysis for *XBP1(s)* or *ATF6^1-373^* , *GRP78*, *PERK* and *LGR5* in cells

treated with different durations of doxycycline 1ug/mL.

**SUPP FIG 6** Expression of XBP1(s) or ATF6^1-373^ results in cell cycle arrest. (A) Representative histogram of cell cycle analysis in LS174T *XBP1(s)*^Tet On^ cells using propidium iodide with on the right the quantification. (B) Representative histogram of cell cycle analysis in LS174T *ATF6*^1-373 Tet On^ cells using propidium iodide with on the right the quantification.All data are shown as means ± SEM. *, P < 0.05, **, P < 0.01, ***, P < 0.001.

**SUPP FIG 7** Knockdown of PERK in XBP1(s) and ATF6^1-373^ expressing cells. (A,B,C) LS174T *XBP1(s)*^Tet On^ cells. (D,E,F) LS174T *ATF6^1-373^* ^Tet On^ cells. (A,D) Protein level of PERK, GRP78 and phosphorylated eIF2α in cells expressing either a shControl, shPERK#1 or shPERK#2. (B,E) Quantitative RT-PCR analysis for *PERK*, downstream target genes *ATF4* and *CHOP* and stem cell marker *LGR5* in the presence of either a shControl or shPERK#2. (C,F) Crystal violet cell viability assay in cells expressing either a shControl, shPERK#1 or shPERK#2. Cells were stimulated with doxycycline or vehicle for 24 hours. All data are shown as means ± SEM. *, P < 0.05, **, P < 0.01, ***, P < 0.001.
