# Supplementary figures and images for: Expression of UPR effector proteins ATF6 and XBP1 reduce colorectal cancer cell proliferation and stemness by activating PERK signaling
Source: Cell Death Dis. 2019 Jun 21;10(7):490. doi: 10.1038/s41419-019-1729-4 (PMC6588629; doi:10.1038/s41419-019-1729-4)

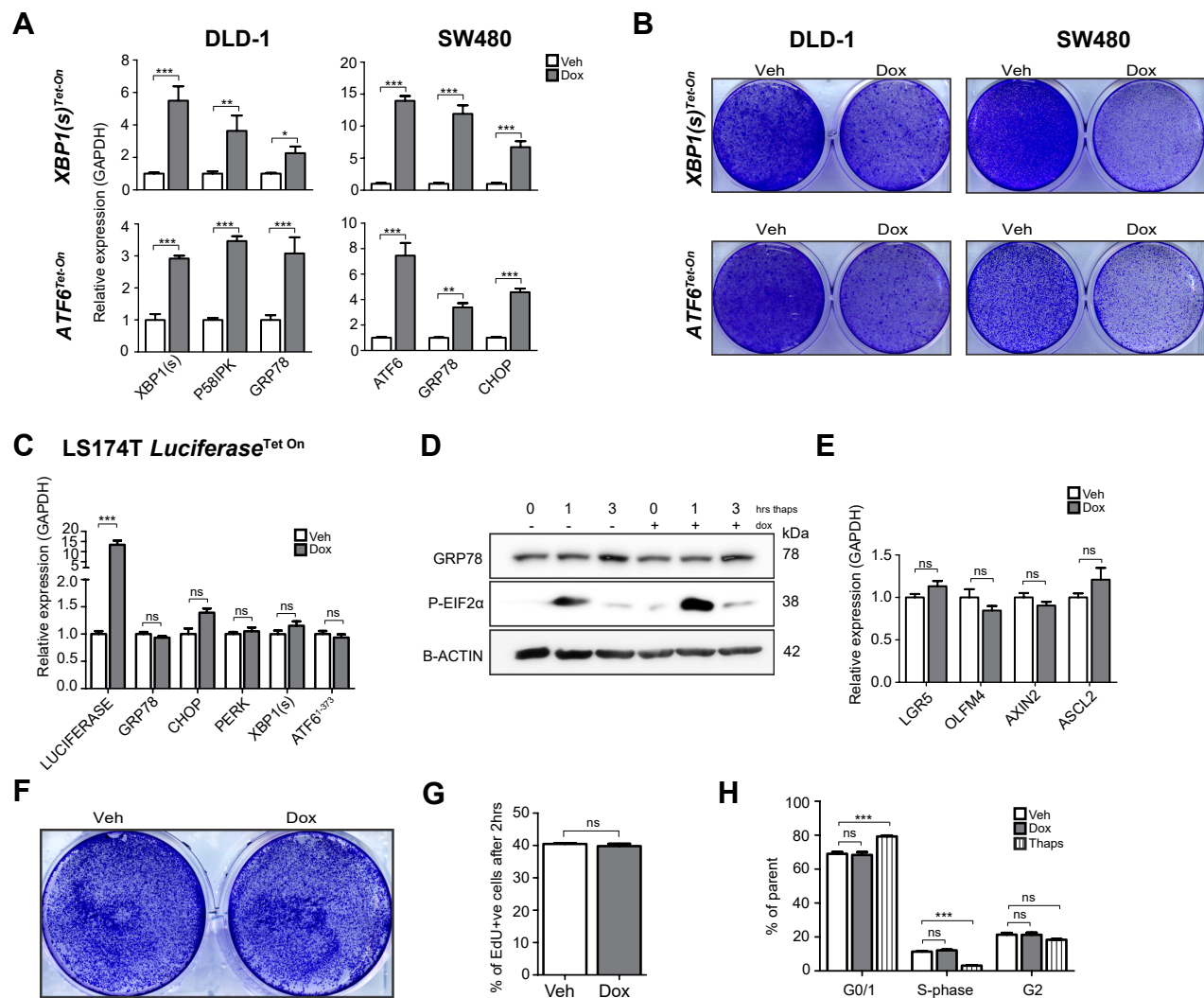

Supplement: Supplementary file 2 — Supp. Fig. 3 [file 41419_2019_1729_MOESM2_ESM.pdf]

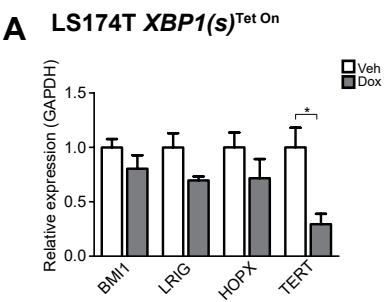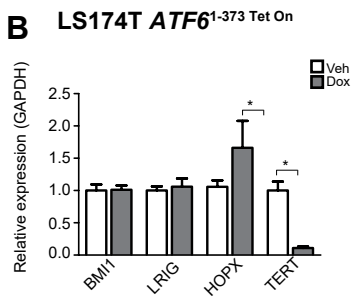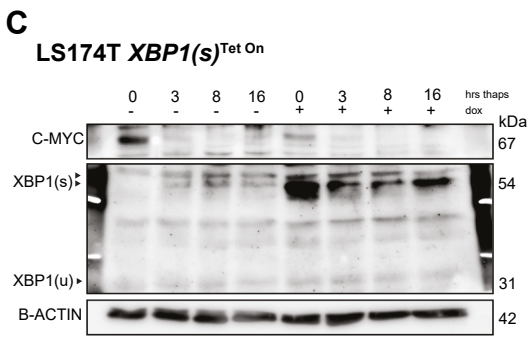

Supplement: Supplementary file 4 — Supp. Fig. 2 [file 41419_2019_1729_MOESM4_ESM.pdf]

**A** LS174T *XBP1(s)*<sup>Tet On</sup>

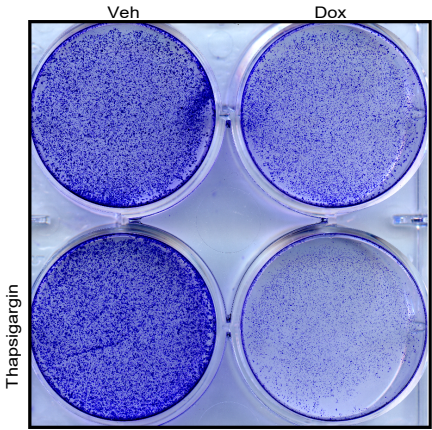

**B** LS174T *ATF6*<sup>Tet On</sup>

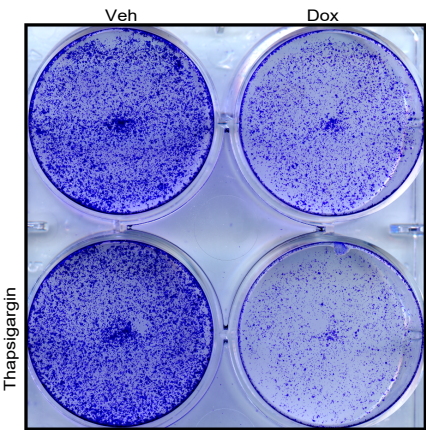

Supplement: Supplementary file 5 — Supp. Fig. 4 [file 41419_2019_1729_MOESM5_ESM.pdf]

**A** LS174T *XBP1(s)*<sup>Tet On</sup>

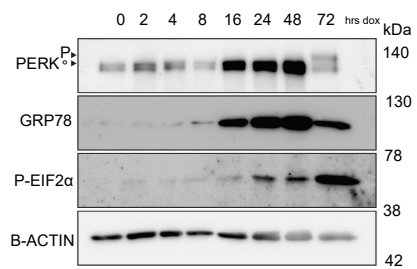

**B** LS174T *ATF6*<sup>1-373 Tet On</sup>

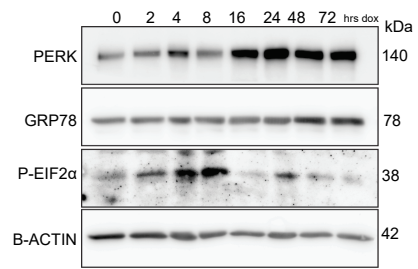

**C** LS174T *XBP1(s)*<sup>Tet On</sup>

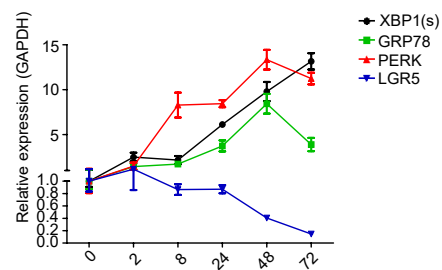

**D** LS174T *ATF6*<sup>1-373 Tet On</sup>

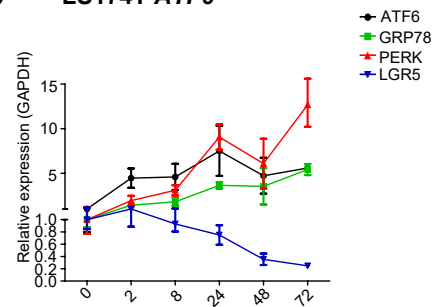

Supplement: Supplementary file 6 — Supp. Fig. 5 [file 41419_2019_1729_MOESM6_ESM.pdf]

**A** LS174T *XBP1(s)*<sup>Tet On</sup>

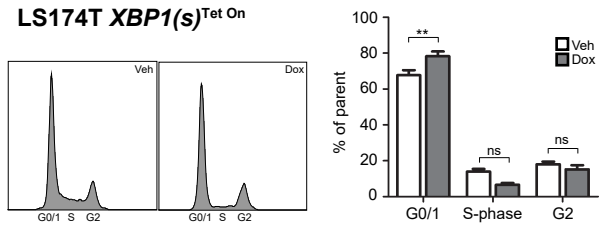

**B** LS174T *ATF6*<sup>Tet On</sup>

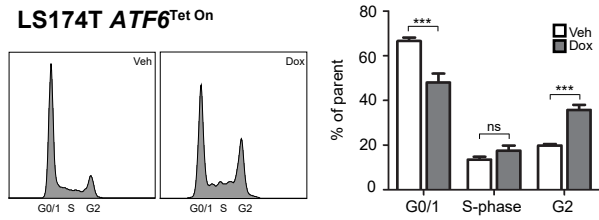

Supplement: Supplementary file 7 — Supp. Fig. 6 [file 41419_2019_1729_MOESM7_ESM.pdf]
