# Supplementary material for: Expression of UPR effector proteins ATF6 and XBP1 reduce colorectal cancer cell proliferation and stemness by activating PERK signaling
Source: Cell Death Dis. 2019 Jun 21;10(7):490. doi: 10.1038/s41419-019-1729-4 (PMC6588629; doi:10.1038/s41419-019-1729-4)
Supplement: Supplementary file 3 — Supp. Fig. 1 [file 41419_2019_1729_MOESM3_ESM.pdf]

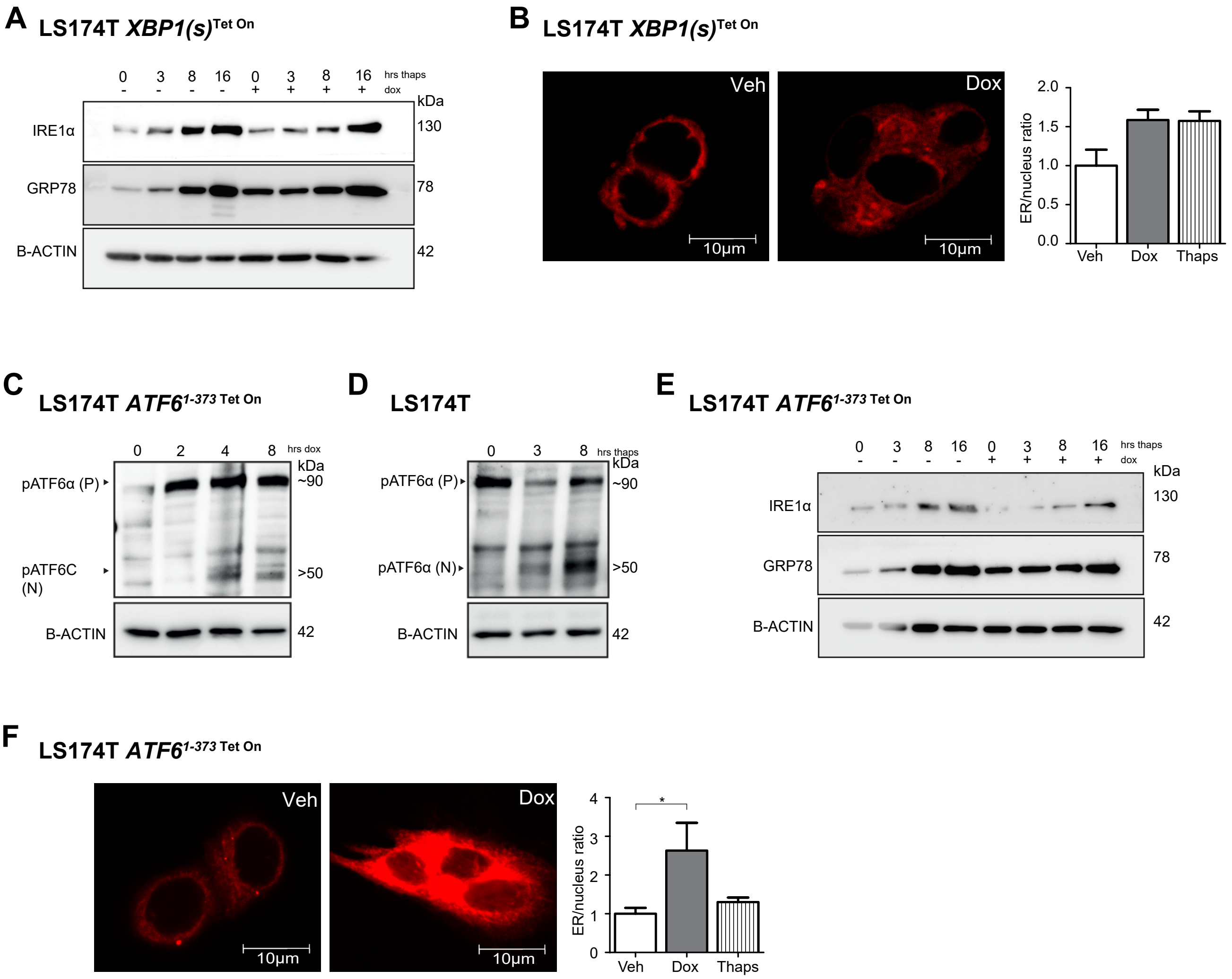

A
LS174T XBP1(s)Tet On
0 3 8 16 0 3 8 16 hrs thaps
- - - - + + + + dox
kDa
IRE1α
130
GRP78
78
B-ACTIN
42
B
LS174T XBP1(s)Tet On
Veh
Dox
10μm
10μm
ER/nucleus ratio
2.0
1.5
1.0
0.5
0.0
Veh
Dox
Thaps
C
LS174T ATF61-373 Tet On
0 2 4 8 hrs dox
kDa
pATF6α (P)
~90
pATF6C (N)
>50
B-ACTIN
42
D
LS174T
0 3 8 hrs thaps
kDa
pATF6α (P)
~90
pATF6α (N)
>50
B-ACTIN
42
E
LS174T ATF61-373 Tet On
0 3 8 16 0 3 8 16 hrs thaps
- - - - + + + + dox
kDa
IRE1α
130
GRP78
78
B-ACTIN
42
F
LS174T ATF61-373 Tet On
Veh
Dox
10μm
10μm
ER/nucleus ratio
4
3
2
1
0
*
Veh
Dox
Thaps
