# Supplementary material for: Expression of UPR effector proteins ATF6 and XBP1 reduce colorectal cancer cell proliferation and stemness by activating PERK signaling
Source: Cell Death Dis. 2019 Jun 21;10(7):490. doi: 10.1038/s41419-019-1729-4 (PMC6588629; doi:10.1038/s41419-019-1729-4)
Supplement: Supplementary file 8 — Supp. Fig. 7 [file 41419_2019_1729_MOESM8_ESM.pdf]

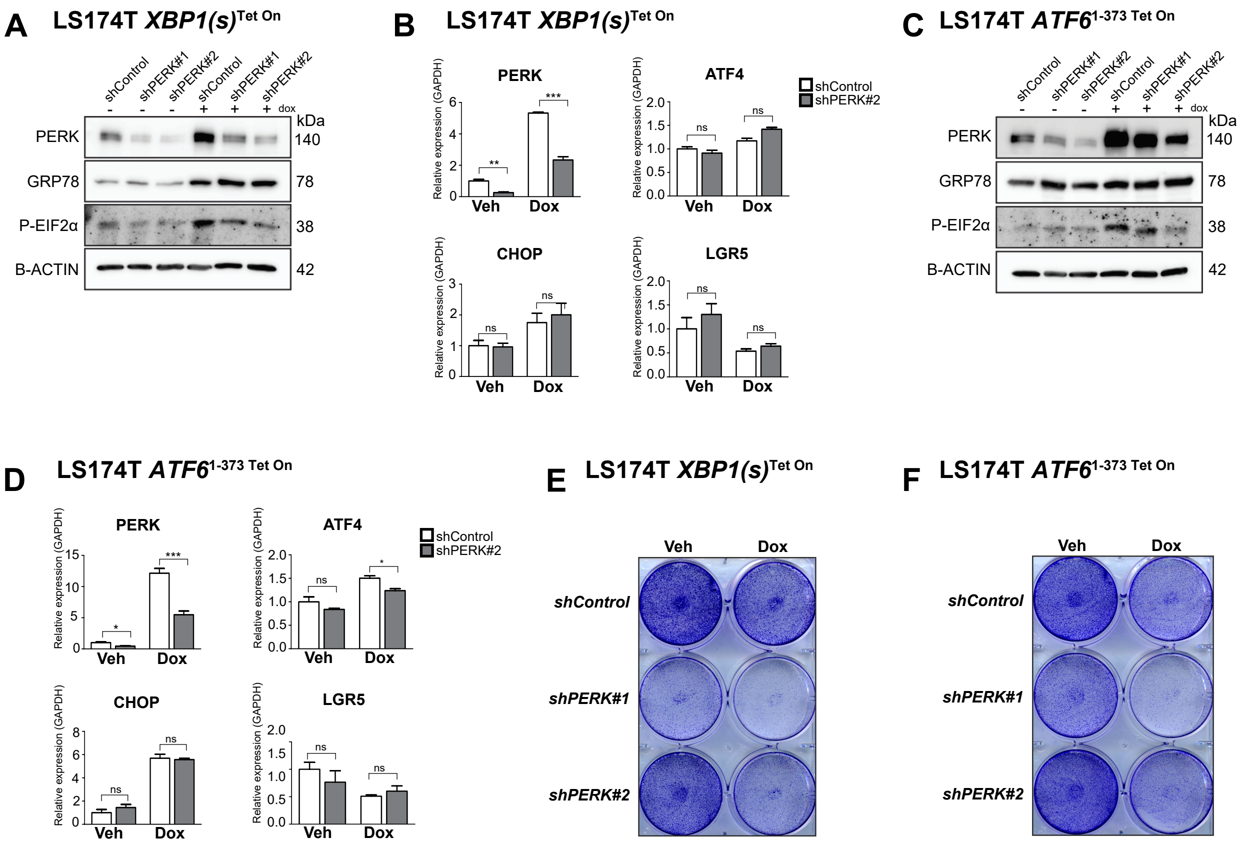

A
LS174T XBP1(s)Tet On
shControl
shPERK#1
shPERK#2
shControl
shPERK#1
shPERK#2
dox
kDa
PERK
140
GRP78
78
P-EIF2α
38
B-ACTIN
42
B
LS174T XBP1(s)Tet On
PERK
ATF4
CHOP
LGR5
Relative expression (GAPDH)
shControl
shPERK#2
Veh
Dox
**
***
ns
C
LS174T ATF6 1-373 Tet On
shControl
shPERK#1
shPERK#2
shControl
shPERK#1
shPERK#2
dox
kDa
PERK
140
GRP78
78
P-EIF2α
38
B-ACTIN
42
D
LS174T ATF6 1-373 Tet On
PERK
ATF4
CHOP
LGR5
Relative expression (GAPDH)
shControl
shPERK#2
Veh
Dox
*
***
ns
E
LS174T XBP1(s)Tet On
Veh
Dox
shControl
shPERK#1
shPERK#2
F
LS174T ATF6 1-373 Tet On
Veh
Dox
shControl
shPERK#1
shPERK#2
